# Interactions and Signal Transduction Pathways Involved during Central Nervous System Entry by *Neisseria meningitidis* across the Blood–Brain Barriers

**DOI:** 10.3390/ijms21228788

**Published:** 2020-11-20

**Authors:** Julia Borkowski, Horst Schroten, Christian Schwerk

**Affiliations:** Pediatric Infectious Diseases, Department of Pediatrics, Medical Faculty Mannheim, Heidelberg University, Theodor-Kutzer-Ufer 1-3, D-68167 Mannheim, Germany; julia.borkowski@medma.uni-heidelberg.de (J.B.); horst.schroten@umm.de (H.S.)

**Keywords:** blood–brain barrier, blood–CSF barrier, host-pathogen interaction, meningeal barrier, meningitis, *Neisseria meningitidis*, signal transduction

## Abstract

The Gram-negative diplococcus *Neisseria meningitidis*, also called meningococcus, exclusively infects humans and can cause meningitis, a severe disease that can lead to the death of the afflicted individuals. To cause meningitis, the bacteria have to enter the central nervous system (CNS) by crossing one of the barriers protecting the CNS from entry by pathogens. These barriers are represented by the blood–brain barrier separating the blood from the brain parenchyma and the blood–cerebrospinal fluid (CSF) barriers at the choroid plexus and the meninges. During the course of meningococcal disease resulting in meningitis, the bacteria undergo several interactions with host cells, including the pharyngeal epithelium and the cells constituting the barriers between the blood and the CSF. These interactions are required to initiate signal transduction pathways that are involved during the crossing of the meningococci into the blood stream and CNS entry, as well as in the host cell response to infection. In this review we summarize the interactions and pathways involved in these processes, whose understanding could help to better understand the pathogenesis of meningococcal meningitis.

## 1. Introduction

*Neisseria meningitidis*, the meningococcus, is a Gram-negative diplococcus exclusively colonizing the human respiratory tract. Thus, humans are the only reservoir and transmission occurs by close contact inhaling respiratory droplets. As Gram-negative bacteria, meningococci have an outer and an inner membrane, between which a thin peptidoglycan layer is located. Meningococci are 0.6–1.0 µm in diameter, with invasive strains expressing a capsule as a major virulence determinant. Further virulence factors include outer membrane proteins as pili, which extend several micrometers beyond the capsule, porins (PorA and -B), and other adhesion molecules [1]. Being a commensal, meningococci do not necessarily induce disease in the carrier. Around 10% of the European population is asymptomatically colonized by *Neisseria,* whereof most carrier isolates are classified nonpathogenic and noninvasive [2]. Genetic polymorphism in the host is likely to be involved in susceptibility and pathogenesis development [3,4]. Multi locus sequence typing (MLST) analyzes sequence variations in DNA fragments of a set of housekeeping genes (*abcZ*, *adk*, *aroE*, *gdh*, *pdhC*, and *pgm*) and helps to identify hypervirulent clones [5], which occur rarely, but cause the majority of disease. Additionally, a bacteriophage in disease-causing clones seems to be associated with increased neisserial virulence [6,7]. Based on the composition of the capsular polysaccharide, *Neisseria* serologically can be classified into 13 serogroups (A, B, C, D, 29E, H, I, K, L, W-135, X, Y, and Z) with 90% of invasive meningococcal infections being related to serogroups A, B, C, W135, and Y [8,9]. Noteworthy, the distribution of meningococcal serogroups varies between countries and regions. Whereas Serogroups C and W are responsible for large amounts of infections in Africa and Latin America, Serogroup B predominantly accounts for disease by *N. meningitidis* in Europe as well as American and Western Pacific regions. Especially in Nordic countries, Serogroup Y is also responsible for a substantial number of cases. Generally, meningococcal disease is highest in the meningitis belt in Sub-Saharan Africa [10].

*N. meningitidis* is a leading cause of sepsis and meningitis in young children (6–24 month) and adolescents [11], and immunization is an important strategy to prevent meningococcal disease. Conjugate vaccines against Serogroups ACWY, C and B are available. Whereas the vaccines against ACWY and C are based on the capsular polysaccharide, the Serogroup B vaccines (Bexsero and Trumenba) contain semiconserved surface components, since the Serogroup B capsular polysaccharide is poorly immunogenic and carries a risk of autoimmunity due to the structural similarity with the human neural cell adhesion molecule [11].

Invasive meningococcal disease (IMD) manifests as only meningitis in around 50% of cases, while other patients can suffer from sepsis, which can culminate in the Waterhouse–Friderichsen syndrome. Clinically, IMD presents with a sudden onset of malaise and unspecific symptoms such as headache, fever, chills, and nausea. Within hours, symptoms progress leading to septicemia and may be accompanied by petechiae and purpura fulminans, meningitis (vomiting and neck stiffness) or shock. Lethality due to meningococcal disease is around 15%, and around 20% of survivors suffer from sequelae [11].

Pharyngeal carriage is a prerequisite for disease. Prior to meningeal inflammation, bacteria have to enter the circulation, persist in the blood with obligatory bacteremia, disseminate, and finally invade the privileged central nervous system (CNS) causing meningeal inflammation [12].

## 2. Blood–Brain Barriers

**Figure 1:**
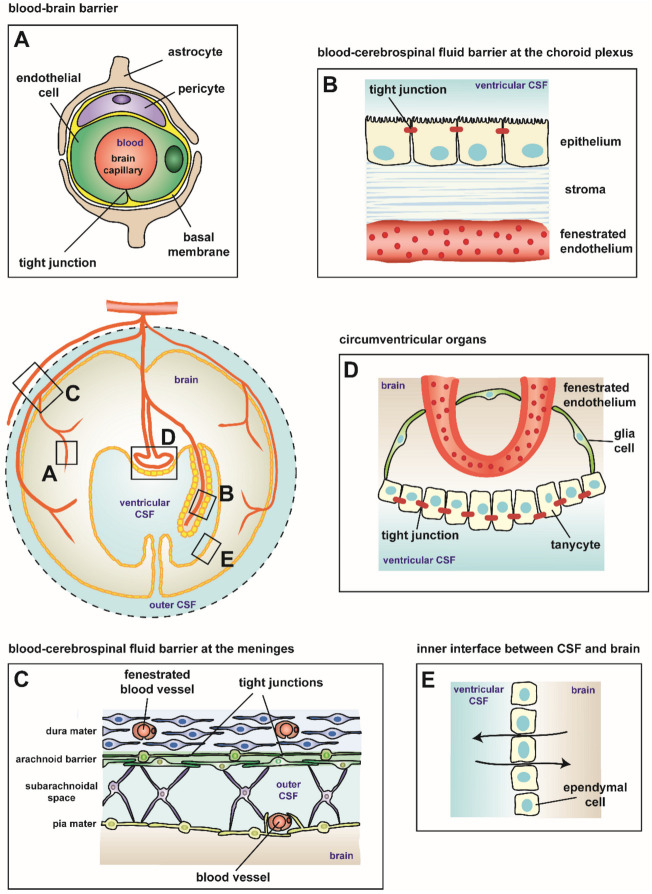
Interfaces between the peripheral tissue and the central nervous system (CNS). (**A**) The blood–brain barrier (BBB) consists of the brain microvascular endothelial cells, connected by tight junctions (TJs), in conjunction with astrocytes and pericytes, and separates the brain parenchyma from the blood. (**B**) The choroid plexus, which is located in the ventricular system, is highly vascularized by a fenestrated endothelium that is placed in the stroma. At the choroid plexus, the blood–cerebrospinal fluid (CSF) barrier (BCSFB) is formed by epithelial cells connected by TJs and separates the blood from the ventricular CSF. (**C**) At the meninges, the dura mater contains fenestrated blood vessels. The arachnoid mater is located adjacent to the dura mater and encloses the subarachnoidal space (SAS) that contains the outer CSF. The BCSFB barrier function of the meninges is formed by the cells of the arachnoid mate, which are connected by TJs. Furthermore, a BCSFB barrier function can be postulated for endothelial cells of pial vessels, which cross the SAS and merge into brain capillaries, but are not covered by pericytes and astrocytic endfeets. (**D**) The blood vessels of the circumventricular organs (CVOs) are fenestrated. The barrier towards the ventricular CSF is formed by TJs between adjacent specialized ependymal cells, termed tanycytes. Glia cells (with TJs) shield against the brain parenchyma. (**E**) Ependymal cells lining the ventricles separate the ventricular CSF from the brain parenchyma. This figure is adapted from [14].

For proper function, the human brain is separated from the rest of the body by specific barriers. A schematic overview of the most relevant interfaces between peripheral tissue and the CNS is given in Figure 1. These are represented by the blood–brain barrier (BBB) localized between the blood vessels and the CNS parenchyma (Figure 1A), the blood–cerebrospinal fluid (CSF) barrier (BCSFB) at the choroid plexus separating the blood from the ventricular CSF (Figure 1B) (reviewed in [13,14]), and the meningeal barrier between the CSF-filled subarachnoidal space (SAS) and the intrameningeal blood vessels (Figure 1C) (reviewed in [12,13,14]). Two additional interfaces in the adult brain should be mentioned: the circumventricular organs (CVOs) barrier formed by tight junctions (TJs) between adjacent tanycytes (Figure 1D) and the inner interface between the CSF and the brain (Figure 1E) [12,14]. The CNS parenchyma (surrounded by the glia limitans) is shielded against the immune system. Ventricular and subarachnoid CSF spaces do not exhibit the same immune privileged status as the CNS parenchyma. The choroidal epithelium and the arachnoid membrane establish an outer barrier, which can be breached by immune cells in case of peripheral inflammation. In the CSF, they face antigen presenting cells (APCs), which may present antigens from the CNS. Immune cells get reactivated and penetrate the inner barrier of the glia limitans subsequently entering the brain parenchyma [13].

### 2.1. Blood–Brain Barrier

Prerequisite for BBB function is a complex interplay of several components based on the neurovascular unit composed of brain endothelium, pericytes, astrocytes, and the basal membranes (Figure 1A).

Brain endothelial cells are characterized by very low pinocytosis rates due to suppression of vesicular activity. However, brain homeostasis ensuring nutrient supply via the blood into the CNS and removal of toxic agents via the contrary direction is maintained by specific membrane transporters, such as glucose transporter 1 (GLUT1) and P-glycoprotein (Pgp) [15]. Free diffusion of ions and molecules via the paracellular route is mostly prevented by TJs closely connecting brain endothelial cells. In parallel, TJs prevent diffusion of proteins within the lipid bilayer and thereby determine cellular polarity. TJs represent a composition of transmembrane proteins, cytoplasmic scaffolding proteins linking the former with the actin cytoskeleton, and signaling proteins. For a long time, the transmembrane proteins claudin-1, claudin-3, claudin-5, claudin-11, and claudin-12 have been considered as relevant TJ components at the BBB, but, due to controversies in several studies, claudin-5 is the only claudin whose expression remains commonly confirmed [16]. Occludin, tricellulin, and MARVEL domain-containing protein 3 (MARVELD3) belong to the tight junction-associated MARVEL protein (TAMP) family and represent a second group of junctional transmembrane proteins. Occludin is likely to be involved in regulating TJ stability and barrier function, but less in TJ assembly. Tricelliulin, as indicated by its name, is localized at tricellular junctions, and its precise function at endothelial TJs remains to be clarified. Junction adhesion molecule (JAM) proteins, a third transmembrane protein group containing JAM-A, JAM-B, and JAM-C, interact with cytoplasmic proteins, thereby providing the linkage to the actin cytoskeleton. JAM proteins regulate cell polarity by interacting with the polarity complex protein Par3. Scaffolding proteins (Membrane-associated guanylate kinase (MAGUK) proteins), such as zonula occludens (ZO)1 and ZO2, connect TJ transmembrane proteins to the cellular cytoskeleton by binding to F-actin. ZO1 is also involved in adherens junction (AJ) assembly, junctional complexes required for TJ formation. In AJ, the intracellular domain of the transmembrane protein vascular endothelial cadherin (VE-cadherin) engages p120 catenin and β-catenin. The anchor protein α-catenin acts as a bridge connecting VE-cadherin with the cytoskeleton by binding to vinculin and ZO1. The nectin-AF-6 complex also contributes to endothelial AJ formation. Platelet endothelial cell adhesion molecule-1 (PECAM-1) belonging to the Ig family is highly expressed at interendothelial junctions maintaining junction integrity [16]. Due to the tight endothelial lining at the BBB, a high transendothelial electrical resistance is generated across the endothelial vessel wall, reaching more than 1500 Ω cm^2^ in capillaries of rats [17].

The luminal side of the vessel wall is lined by a glycocalyx composed of a carbohydrate-rich mesh of anionic polymers representing already a first line of barrier for blood borne pathogens. The basolateral cell side faces the endothelial basement membrane composed of extracellular matrix (ECM) proteins (a network of type IV collagen, α4 and α5 laminins, nidogen, heparan sulfate proteoglycans and some glycoproteins) secreted by both endothelial cells and pericytes. The basement membrane includes embedded pericytes (endothelial cell/pericyte ratio between 1:1 to 3:1), together with smooth muscle cells covering the endothelial cells, and builds the tunica media. Unique to brain endothelial cells is a second basement membrane, which includes α1 and α2 laminins secreted by adjacent astrocytes, and is named parenchymal basement membrane. Together with astrocytic endfeet, the parenchymal basement membrane represents a thin physical barrier, the glia limitans. At the level of post-capillary venules, a small space is generated between the two membranes called perivascular space. At the capillary level, the two membrane leaflets merge inseparably (the BBB structure has recently been reviewed in [16]).

Most studies of pathogens penetrating the CNS via the endothelial route focus on brain microvascular endothelial cells exhibiting a tight barrier. Endothelial cells of brain post-capillary venules and of subpial and subarachnoidal veins located in close vicinity to the SAS, however, exhibit less barrier function than the endothelial cells of the brain parenchyma. Due to their “leaky” interendothelial junctions, they are more likely to be the primary site of passage of pathogens into the CSF [18]. In the case of passing through subpial and subarachnoidal veins, the bacteria face the meningeal barrier, whose structure and interaction with meningococci is further addressed below.

### 2.2. Blood–Cerebrospinal Fluid Barrier at the Choroid Plexus

The BCSFB faces circulating fluids on both sides, the blood on the basolateral side and the CSF on the apical side. The barrier itself is formed by choroidal epithelial cells, which surround a highly vascularized stromal tissue, together inclosing the CSF-filled ventricles (Figure 1B). Four choroid plexus exist in the brain being located at the two laterals and the third and fourth ventricles [13].

The choroid plexus epithelial cells secrete vascular endothelial growth factor (VEGF), thus controlling formation and maintenance of endothelial fenestrae (involving plasmalemma vesicle-associated protein (Plvap)). Fenestration allows blood components to easily get in contact with the stromal tissue. Additionally, the epithelial cells produce CSF. Consistently with being located at the interface of two fluids, cells exhibit basolateral infoldings as well as apical microvilli, thereby increasing the surface area and allowing substantial exchange [13]. Transport processes across the barrier, however, are strictly regulated. ABC transporters as well as solute carrier (SLC) transporters monitor the return of lipophilic molecules back to the blood and the transport of ions and amino acids into the CSF, respectively. Claudin-2 and aquaporin 1 ensure paracellular and transcellular water flow from blood to the CSF. Paracellular permeability across the epithelial cells is limited due to the presence of TJ complexes localized at the apical lateral cell side. AJ are localized basolaterally of TJ. Together both junctional complexes determine cellular polarity [16].

Due to the junctional sealing, choroidal epithelial cells produce a strong transepithelial electrical resistance (TEER), which, however, is lower than TEER values generated at the BBB. AJs at the BCSFB are composed similar to the BBB with epithelial cadherin (E-cadherin) replacing VE-cadherin. E-cadherin binds to p120- and β-catenin, which in turn bind to α-catenin linking E-cadherin to the actin cytoskeleton. Several claudins (claudin-1, claudin-2, claudin-3, claudin-9, claudin-10, claudin-11, and claudin-19) have been reported to be expressed at the choroid plexus TJs. Occludin and ZO-1 as well are involved in TJ formation. JAM-A and JAM-C, only at the choroid plexus of the fourth ventricle, are present at the TJs at the BCSFB. Similar to endothelial TJ complexes, several studies report controversial results, thus the contribution of each component requires ongoing clarification. Recently, a protein called Alix has been shown to be highly expressed at the TJ at the BCSFB, leading to massive tight junctional disorganization and hydrocephalus, if absent [13,16].

Due to its strategic interfacial localization, the choroid plexus integrates signals from both the blood and the brain, and dynamically responds via modulation of its transcriptome, proteome, and secretome acting as an important sensor of changing conditions or stimuli. Furthermore, the choroid plexus has an important role during neuroimmune surveillance and is associated with several types of immune cells of both, the adaptive and innate immunity. Epiplexus Kolmer cells reside on the apical/CSF-facing side. On the vascularized stromal/basolateral side, major histocompatibility complex (MHC) class II expressing macrophages, dendritic cells, and myeloid progenitor cells are located directly being exposed to blood components. In contrast to the CNS parenchyma, which is free from cells of the adaptive immune system, 150,000–750,000 immune cells are present in the CSF, mostly representing T-memory cell populations. Additionally, those immune cells likely enter the CNS via the choroid plexus route, thus the choroid plexus holds an important role in regulating the balance of immune cell trafficking into the CNS during immune surveillance and CNS pathology (choroidal BCSFB structure and function have recently been reviewed in [13,16]).

### 2.3. Blood–Cerebrospinal Fluid Barrier at the Meninges

The meninges cover the CNS, brain, and spinal cord. The dura mater is the most peripheral part facing the skull bone composed of dense collagen fiber bundles containing arteries, veins, and lymphatics. Located adjacent to the dura is the arachnoid mater enclosing the underlying CSF-filled SAS (Figure 1C). Trabeculae, which represent collagen bundles coated by leptomeningeal cells, extend from the inner arachnoid layer across the SAS to join the pia mater or suspending subarachnoid blood vessels in the SAS. The pia mater is the most inner part tightly facing the brain parenchyma and closely surrounding vessels following their way penetrating the CNS parenchyma. Together, pia and arachnoid mater form the leptomeninges composed by leptomeningeal cells. The BCSFB barrier function of the meninges is strictly formed by the arachnoid mater, shielding the CSF from dural vessels, which exhibit fenestrae. Thus, leptomeningeal cells of the outer layer of the arachnoid, where it abuts the dura, are connected by TJ. Desmosomes join leptomeningeal cells of the arachnoidea main body and less frequently of the pia. Pial cells coating trabeculae, arteries, and veins in the SAS are connected by small gap junctions. Given the fact that bacterial meningitis predominantly represents an inflammation of the leptomeninges, this barrier may play a more relevant role than supposed to [19,20]. An additional BCSFB barrier function can be postulated for the endothelial cells of pial vessels crossing the SAS following penetrating arterioles and merging into brain capillaries, thus representing a continuity with the microvascular endothelial cells of the BBB (meningeal BCSFB structure has been reviewed in [12,16]). However, the pial endothelium is not covered by pericytes and astrocytic endfeets.

Similar to the choroid plexus, the arachnoid contributes to CNS immunity regulation due to a high number of MHC class II expressing myeloid cells strategically positioned on both sides of the membrane. On the dura-facing side, macrophages and dendritic cells are located, and the CSF-facing side harbors resident macrophages working as scavenger and possibly presenting antigens to entering T-cells [13].

## 3. Neisserial Interactions with the Host

### 3.1. Colonization as a Prerequisite for Meningococcal Disease

A prerequisite for meningococcal CNS entry is bacteremia. Since IMD commonly manifests as meningitis or fulminant sepsis, bacteremia during meningococcal meningitis pathogenesis seems to be asymptomatic [12]. Once transferred to a new host, meningococci get in contact with the human nasopharyngeal epithelium. Initially, only a few individuals attach to the epithelial cells. Microcolonies with a high number of interacting bacteria develop by bacterial replication [18]. As the human respiratory tract is the only natural reservoir of meningococci, dissemination to new colonization sites within the same host or to a new host takes its run from here. A regulated detachment process releasing individual bacteria from microcolonies favors their dissemination. As shown in vitro with human cell lines, the underlying mechanism is based on a posttranscriptional modification during which a transferase adds phosphoglycerol to type IV pili (Tfp). Thereby, the Tfp dependent interbacterial contacts in the bacterial aggregations are limited and dissemination is promoted [21]. Initial attachment at the new colonization site of human cells or organ cultures in vitro is also mediated by meningococcal Tfp [22,23,24,25,26], the most important adhesins of encapsulated strains passing through the capsular polysaccharide and extending from the bacterial surface. Other adhesins remain concealed by the capsule.

CD46, a regulatory protein of the complement system, has been identified as an epithelial surface receptor for pilated meningococci in vitro [27]. Additionally, a human CD46 transgenic mice model demonstrated that upon intranasal challenge those mice were susceptible to IMD, since the bacteria could cross the BBB efficiently [28]. However, inconsistent with CD46 being a primary Tfp receptor during adhesion is its basolateral expression [29,30]. Due to several other controversies the role of CD46 during meningococcal pathogenesis remains still unclear [31,32,33,34].

Tfp dependent adhesion to human epithelial cell lines in vitro elicits rapid localized rearrangements in the cellular cortex, which are accompanied by the formation of specific molecular complexes, called cortical plaques, underneath the attached bacterial colonies. These structures result from the re-localization of specific proteins in the plasma membrane, including components of the cortical cytoskeleton and integral membrane proteins. Thus, the Tfp contact induces localized actin polymerization and the recruitment of ezrin, the second cortical cytoskeleton component, from microvilli-like protrusions to the attachment sites. Ezrin serves as a linker between the actin cytoskeleton and transmembrane proteins and subsequently promotes the recruitment of Ezrin binding proteins, which are redistributed from their original localization, mainly at cell protrusions and at lateral cell–cell junctions, to form clusters beneath bacterial attachment sites [35,36].

In human pharyngeal organ cultures the localized actin polymerization leads to the formation of microvilli-like protrusions closely accommodating the bacterial colonies and in consequence to their internalization [37]. Following this initial Tfp-dependent adhesion step, bacteria get in close contact (intimate adhesion) with the host cell. To enable intimate adhesion, pili get retracted and bacteria become tightly associated with the host membrane, as shown with human epithelial cells in vitro [22]. Microvilli disappear during intimate adhesion and the cell surface becomes denuded from microvilli at the bacterial attachment sites, while meningococci at the actin polymerization sites remain present. Thus, Tfp may act as sensors, mediating initial cell contact, but upon contact get downregulated. Upon contact with human epithelial cells in vitro, capsule synthesis is also repressed, as the capsule may hinder intimate adhesion as well, thereby unmasking surface structures that may be involved in intimate adhesion [38]. Upon this intimate contact, meningococci may cross the epithelial respiratory barrier following a transcellular route involving the microtubule network without affecting barrier integrity. Initial capsule and Tfp expression, however, is required for crossing and capsular expression is important for intracellular survival in vitro [34,39]. With the crossing of the nasopharyngeal barrier and meningococci reaching the blood stream, carrier status is left and pathology begins.

However, meningococci may not necessarily have to use the blood circulation to reach the meninges. An alternative route has been described for intranasally infected mice that revealed epithelial lesions and junctional alterations at the nasal epithelium in particular at the olfactory epithelium, upon infection. The bacterial distribution suggested that meningococci directly passed from the nasopharynx to the meninges via the olfactory nerve system [40].

#### Minor Adhesins and Invasins Involved during Interaction with Epithelial Cells

Capsule downregulation unmasks minor adhesins and invasins, such as the opacity proteins Opc and Opa as well as NadA (involved in adhesion/invasion to/in epithelial cells), NhhA and App (autotransporters involved in adhesion to epithelial cells), and MspA (autotransporter involved in adherence to epithelial and endothelial cells) that together with App has been shown in vitro to be transported to the nucleus, where it proteolytically cleaves the core histone H3 [41], and HrpA (two partner secretion system) that might be involved in adhesion to epithelial cells [42].

Opa are classified into two categories. A small group of variants bind to heparin sulfate proteoglycans (HSPGs) and ECM proteins (fibronectin and vitronectin), most variants, however, bind to carcinoembryonic antigen-related cell adhesion molecules (CEACAMs). In contrast to CEACAMs, HSPG receptors are not expressed on the apical surface of polarized epithelia, such as the nasopharyngeal epithelium, thus interaction with HSPG receptors is not involved in apical adherence [42]. As described, pilus retraction upon initial attachment mediates intimate contact favoring interaction of Opa and apical expressed CEACAM leading to engulfment and transcytosis thereby entering subepithelial spaces. CEACAM1-triggered penetration of Opa-expressing meningococci of the nasopharyngeal epithelium was also confirmed in a CEACAM1-humanized mice model [43]. Binding to CEACAM may also suppress toll-like receptor 2 (TLR2)-dependent innate responses in epithelial cells by bacterial trapping. Thereby, TLR2 initiated, NFkB-dependent inflammatory responses are reduced, as shown in in vitro cultures of human primary bronchial epithelial cells obtained from normal human volunteers [44]. Interaction with CEACAMs may further affect several immune functions (CEACAM Opa interaction has been reviewed in [42]).

Opc can interact with epithelial cell lines in vitro via HSPGs, without the need of human serum factors vibronectin and fitronectin as described for Opc-dependent binding to endothelial cells, inducing signaling and subsequent internalization [45,46,47].

### 3.2. Neisserial Survival in the Blood

Meningococci have evolved several strategies to evade innate immune defense in the blood. Besides the expression of several factors that favor their survival in the blood (TspB [48], TbpAB [49], LbpAB [50,51], HpuAB [52], HmbR [53], NHBA [54], Ig-binding protease [55], PorA [56], NspA nd PorB3 [57], fHbp [58], and others [59,60]), strategies are mostly based on modulation of surface components. The capsular polysaccharide and lipopolysaccharide (LPS) exhibit characteristic antigenic variation enabling meningococci to avoid antibody deposition, complement-mediated killing, and phagocytosis [12]. Horizontal gene transfer (intragenomic recombination due to natural competence) in the capsule operon can provoke capsule switching [61,62,63]. In addition, exchange of genetic alleles (intergenomic recombination of gene segments due to availability of several gene copies in the genome) in the *opa* genes [64,65] or *pil* genes [66,67] creates new variants. Antigenic variation can additionally be achieved by phase variation. Addition or reduction of repetitive sequences due to single strand mispairing (ssm) during replication can modulate expression levels [68,69]. Ssm outside the open reading frame (ORF) can affect transcriptional efficiency as well as posttranslational events or binding of regulatory proteins. Ssm inside the ORF may result in on/off switching of genes such as the capsule [70], LPS [71] or *opa* genes [72]. Reversible exchange of mobile insertion elements inside the *siaD* gene also effects capsule variation [73].

Besides variation, evasion can also be achieved by molecular mimicry of host structures due to the addition of sialic acid to the capsular polysaccharide [74] or the LPS [71,75]. The serogroup B capsule itself is already poorly immunogenic due to the structural similarity to the human neural cell adhesion molecule [11]. Furthermore, posttranslational modification by attachment of phosphorylcholine may enable interaction with the PAF-receptor and is likely to be involved in immune evasion as well [9].

Vascular colonization of peripheral vessels that play a relevant role during meningococcal induced purpuric skin lesions proceeds in the same way as is described for cerebral endothelial cells below [76,77,78,79].

### 3.3. Neisserial Interactions with the Host during CNS Entry

Via the blood stream, the bacteria reach the anatomical structures described in Figure 1. The most relevant barriers during CNS invasion by *N. meningitidis* are the BBB, the BCSFB at the choroid plexus, and the BCSFB at the meninges. The interactions of meningococci with host cells at these barriers and the signaling pathways involved are discussed in the following.

#### 3.3.1. Neisserial Interactions with the Blood–Brain Barrier

Endothelial cells of the BBB (Figure 1A) have been prevalently considered to be the primary entry site of meningococci into the CNS with a possible draining into the SAS via the glymphatic pathway [18,80]. The bacteria engage in first contact with the luminal side of the brain microvasculature. Several in vitro models have been established to study meningococcal interaction with brain endothelial cells [81]. A humanized mouse model implanted with human skin containing human dermal vessels has been established to analyze interaction with dermal microvascular cells [82], which has been shown to be similar to cerebral microvascular cells [76].

**Figure 2:**
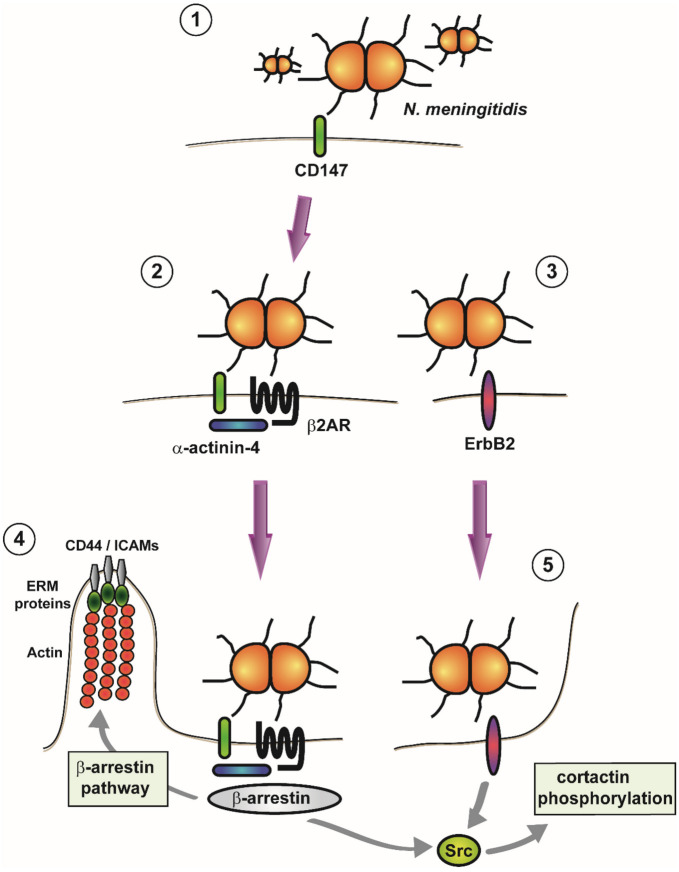
Adhesion of *Neisseria meningitidis* to the endothelium. (**1**) In an initial step, meningococci adhere to the immunoglobulin superfamily member CD147 on the surface of host cells. (**2**) Subsequently, the G protein coupled β2-adrenergic receptor (β2AR) forms heteromeric complexes with CD147 that are stabilized by α-actinin-4. (**3**) Adhesion of *N. meningitidis* also leads to the recruitment of the ErbB2 receptor. (**4**) Activation of β2AR in return causes activation of the β-arrestin pathway, leading to plasma membrane remodeling and the recruitment of ERM proteins (Ezrin and Moesin), together with ERM-binding transmembrane proteins (CD44 and ICAMs) and cortical actin (cortical plaque formation). Src is also recruited and activated. (**5**) Association of *N. meningitidis* with ErbB2 leads to recruitment and activation of Src, which in turn causes phosphorylation of cortactin (which triggers the formation of membrane protrusions).

The steps participating during adhesion of *N. meningitidis* to endothelium are summarized in Figure 2. The host cell receptor responsible for initial meningococcal attachment to both, peripheral and cerebral, endothelium has been remained obscure for a long time. Several receptors have been reported to be the Tfp interacting receptor such as CD46 [27,28,31,33], the Laminin receptor [83], or the platelet activating factor receptor (the latter, however, on human epithelial airway cells) [84]. Recently, however, CD147, a member of the immunoglobin superfamily expressed on brain capillaries, has been identified to be a critical receptor for Tpf (PilV and PilE)-dependent adhesion. Receptor interaction has been verified in vitro to human brain and peripheral endothelial cells and to induced pluripotent stem cell-derived brain endothelial cells, in vivo in a mice model with xenografted human skin containing dermal microvasculature, and ex vivo in human brain tissue explants [77,85].

As shown in vitro using endothelial cell lines, consecutively upon the initial adhesion to CD147 the G protein coupled β2-adrenergic receptor (β2AR) is recruited beneath meningococcal colonies and both receptors form highly ordered heterooligomeric complexes scaffolded by α-actinin-4 increasing the binding strength of meningococci under shear stress [86]. Activation of β2AR in a Tfp dependent manner results in the activation of the β-arrestin pathway with a local remodeling of the plasma membrane due to the recruitment of ezrin and moesin (ERM proteins), as well as ERM-binding transmembrane proteins, such as CD44, ICAM-1/-2, cortical actin, and the activation of the Rho GTPases (Rho and Cdc42) [87,88]. Src kinase is also recruited and activated [88,89]. Together, these events lead to the formation of specific protein clusters (cortical plaques) accompanied by local actin polymerization, finally promoting the formation of membrane protrusions highly enriched in the recruited proteins at the bacterial attachment sites [88]. Those microvilli-like protrusions stabilize the colonies and protect them against hemodynamic forces. Ezrin recruitment and sheer stress resistance are based on the presence of PilV [90]. A genetic polymorphism in the β2AR has been identified to be associated with increased susceptibility to bacterial meningitis [3].

Most of the transmembrane proteins, which are sequestrated in the cortical plaques at neisserial attachment sites, are key players in leukocyte adhesion and guidance including CD44, ICAM-1, and VCAM-1. Their titration towards bacterial colonies prevents the formation of leukocyte docking structures resulting in reduced leukocyte contacts and their subsequent diapedesis. Consequently, in a human bone marrow endothelial cell line, *Neisseria* downregulates the inflammatory response upon infection by interfering with leukocyte-endothelial interaction [91].

Work with primary endothelial cells and endothelial cell lines has shown that Tfp-mediated adhesion also induces ErbB2 receptor recruitment to attachment sites and its subsequent phosphorylation [76,92,93]. Active ErbB2 recruits Src kinase and results in its subsequent activation, which in turn leads to the phosphorylation of cortactin triggering the formation of protrusions [93]. ErbB2 is recruited to the site, where cortical plaques develop, but the ErbB2/src pathway is not involved in their formation (ezrin recruitment and actin polymerization). Ezrin recruitment occurs independent of Rho and Cdc42 [87]. Actin and cortactin polymerization, however, depend on Rho and Cdc42 activation [87,93]. Bacterial internalization is only partially affected upon Rho and Cdc42 inhibition [93], suggesting that the bacteria-induced reorganization of the actin cytoskeleton in cooperation with the ErbB2/Src and the GTPase dependent signaling pathway results in an efficient bacterial internalization [93]. ErbB, β-arrestin, and integrin signaling can activate the mitogen-activated kinase (MAPK) pathways in vitro [94,95], which have been shown to be activated upon neisserial infection affecting internalization and cytokine release in a human brain microvascular endothelial cell line [96]. Phosphoinositide3-kinase/Rac1 signaling dependent cortactin recruitment triggered by LPS has also been reported to be involved in meningococcal invasion of endothelial cells in a human bone marrow endothelial cell line [97].

In endothelial cell lines, protrusional engulfment of meningococci can lead to internalization into intracellular vacuoles [87]. Similarly, subsequently to invasion into human brain microvascular endothelial cells, meningococci are found inside of *Neisseria*-containing vacuoles [98]. These vacuoles correspond to early and late endosomal and/or lysosomal compartments as demonstrated by colocalization with the transferrin receptor and LAMP-1, respectively. The bacteria replicate in the vacuoles and are protected by the capsule. It has been suggested that internalization into intracellular vacuoles and the intracellular replication could play a role during barrier crossing [87,98]. However, determination of the mechanisms during a putative transcellular crossing of the BBB by meningococci requires further investigation.

Despite the data pointing to a transcellular route through endothelial cells, there is also evidence that meningococci cross the endothelium using the paracellular route via gaps between cells due the delocalization of junctional proteins (AJ proteins such as VE-cadherin, p120 catenin, and β-catenin as well as TJ proteins such as ZO1, ZO2, and claudin-5). Results in human brain microvascular endothelial cell lines show that this event could occur upon the Tfp-dependent recruitment of the polarity complex (Par3/Par6/PKCζ) underneath meningococcal colonies. Polarity complex recruitment is induced by binding of *N. meningitidis* to host cell receptors, followed by recruitment of ezrin, clustering of transmembrane proteins and activation of the GTPase Cdc42. Tearing apart the proteins from the junctional complexes and sequestrating them at the attachment sites leads to an increased barrier permeability that promotes a paracellular crossing of the BBB [99]. Activation of Matrix Metalloprotease (MMP) 8 proteolytically cleaving occludin, an endothelial TJ protein, as well as favors opening of the paracellular route for meningococcal crossing of the BBB [100].

Besides cellular reorganization based on intracellular protein interactions upon infection, human brain microvascular endothelial cells exhibit an adjusted transcriptional profile when infected with *N. meningitidis* in vitro. A transcriptional increase of genes encoding for proteins involved in bacterial adhesion and invasion, apoptosis, and cell adhesion, as well as in downstream signaling of integrins and corresponding negative regulators has been observed. In addition, genes involved in cytoskeleton reorganization are regulated. Almost 50% of the regulated genes depend on capsule expression [101].

During fulminant meningococcal disease with purpura fulminans, meningococci not only adhere and cross brain endothelial cells, but also breach the vascular wall of peripheral endothelial cells, leading to the characteristic hemorrhagic lesions of the skin, as shown in a model employing human skin grafted SCID mice [78]. Recruitment of the polarity complex finally opening cell–cell contacts by tearing away the junctional proteins has been shown to be a common feature upon neisserial infection in primary peripheral and cerebral endothelial cells [76].

Penetration of the nasopharyngeal epithelium and the endothelium in the periphery and at the BBB, however, involves two different signaling pathways [76]. This can be explained by the different local ambient conditions (normally slow shear stress in the nasopharynx besides coughing, and varying, but steadily existent, shear stress in the blood) and the intention behind the colonization of the respective niche (crossing of the nasopharyngeal barrier/spreading to new attachment sites, and crossing of peripheral and cerebral endothelial barriers/vascular colonization against shear stress) [76,102]. Common and differing features in signaling induced upon the attachment of meningococci to epithelial and endothelial cells are displayed in Table 1.

Pilated and encapsulated phenotypes are isolated from blood and CSF [12]. Minor adhesins located in the outer membrane are mostly concealed due to their subcapsular localization. A possible scenario similar to colonization of the nasopharyngeal epithelium would include retraction of Tfp and a concomitant downregulation of the capsule, thereby exposing adhesins and ensuring a close contact [12]. Recently, several thus far unknown potential meningococcal ligands interacting with human brain microvascular endothelial cells have been recovered and identified upon infection [103]. Non-Tfp-mediated interactions become relevant in vitro under inflammatory conditions or during receptor clustering, both cases leading to a high degree of receptor density [104,105,106,107,108].

Thus far, the most prominent role during endothelial interaction probably is probably played by the subcapsular invasin Opc that in vitro interacts with the extracellular matrix proteins vitronectin and fibronectin [105,109,110], which occur abundantly in human serum. However, it preferentially binds to sulfated tyrosine residues of the activated form of human vitronectin, but not to native vitronectin [110]. Via a sandwich mechanism with vitronectin and fibronectin acting as molecular bridges, Opc binds to integrins (αVβ3-integrin/vitronectin and α5β1-integrin/fibronectin) on the apical surface of endothelial cells. Thereby, meningococci are linked to the integrins subsequently leading to the formation of a trimolecular complex [105,109]. Integrin signaling is dependent on adapter molecules, such as protein tyrosine kinases, due to their lack of intrinsic enzymatic activity [111]. In a human brain microvascular endothelial cell line, Src kinase has been shown to be activated Opc-dependently, linking the cytoplasmatic integrin tails to the actin cytoskeleton promoting the meningococcal uptake by actin rearrangements [112]. In addition to Src, Opc-dependent meningococcal internalization depends on focal adhesion kinase (FAK) and cortactin [113]. Once internalized Opc can interact in vitro with the intracellular cytoskeletal protein α-actinin, a modulator of signaling pathways and cytoskeletal functions, thereby facilitating the passage across endothelial barriers [114]. Opc is suggested to be relevant for crossing of cerebral endothelial cells, since Opc-lacking strains of the ET-37/ST11 clonal complex can affect clinical disease profile in being more virulent to cause serious septicemia than meningitis [68,105,115,116,117].

Opc and Opa can not only function as adhesins/invasins, but also act as cyclomodulins, known to hijack cell cycle check points, to establish bacterial infection. In human brain endothelial cells in vitro, meningococcal infection reveals an accumulation of brain endothelial cells in the S-phase (DNA replication) in parallel with a decrease of cells in G2/M phase (gap preparing cells for division/mitosis) leading to cell cycle arrest [118]. Cell cycle arrest has also been observed in nasopharyngeal epithelial cells in vitro. However, epithelial cells exhibited an arrest in G1-phase (gap between DNA replication and mitosis), which manifested in an accumulation of those cells in G1. In both brain endothelial and nasopharyngeal epithelial cells, cell arrest correlated with an accumulation and increased nuclear localization of CKI p21^WAF1/CIP1^ a cell cycle inhibitor, known to slow cell cycle and to induce cell cycle arrest in G1-, G2-, or S-phase when overexpressed [118,119].

Additionally, Opc is required to induce specialized ceramide-enriched membrane domains, which provide a proper receptor and signaling molecule clustering. Opc expressing meningococci bind to HSPGs followed by activation of phosphatidylcholine specific phospholipase C (PC-PLC), leading to the activation of acid sphingomyelinase (ASM) in human brain microvascular endothelial cell lines. ASM metabolizes sphingomyelin into ceramide and phosphorylcholine. Ceramide release results in the formation of ceramide-enriched raft domains, trapping receptors and signaling molecules such as ErbB2 and cortical plaques proteins, thereby favoring bacterial entry [120].

#### 3.3.2. Neisserial Interactions with the Blood–Cerebrospinal Fluid Barrier at the Choroid Plexus

In contrast to interactions of meningococci with the BBB, interactions with the BCSFB at the choroidal epithelial cells have been less investigated, likely due to the fact that meningococci exclusively interact with human cells and the lack of appropriate human BCSFB models. The development of those models is definitely of high relevance, since in a previous case of fulminant meningococcemia meningococci were found in particularly high numbers adhering to endothelial cells of capillaries of the choroid plexus and to a lesser extent to endothelial cells of capillaries of the meninges. Since the patient died early during disease progress before an acute inflammation of the meninges, but bacteria could already be recovered from the CSF, it was likely to find bacteria still attached to the structures they used for their entry [121]. This can be a problem when disease has progressed, since bacteria have spread and secondary effects cannot be excluded. However, the relevance of the choroid plexus as a CSF entry route often is understated with the argument that no bacteria were found adhering to or between the choroidal epithelial cells. The bacterial crossing through the meningeal capillaries is considered as the more likely scenario, although bacteria inside of meningeal endothelial cells or between them has not been addressed in this study. Another study could indeed demonstrate meningococci localized inside of brain endothelial cells of an infected patient, but again the authors found meningococci also attached to and inside of choroidal endothelial cells [122]. The choroid plexus being a possible entry site is also supported by the findings of high concentrations of meningococci in blood vessels of the choroid plexus and also, even if sporadic, in the choroid plexus epithelium [123]. Interacting with several components of the brain barriers, no final conclusion regarding the exact invasion site can be drawn [122].

A fact that has to mentioned and is part of the discussion in the context of the choroid plexus being a meningococcal entry site into the CSF is that, in consequence of a penetration via the BCSFB, an inflammation of the plexus (so called plexitis) or of the ventricles and their surrounding ependyma (ventriculitis) would be expected. However, this is rarely observed in clinical practice and if so, then rather as an associated secondary complication. Some bacterial induced ventriculitides, however, may be under diagnosed since magnetic resonance tomography (MRT) is not regularly performed in meningitis patients [124].

In conclusion, the role of the choroid plexus as a meningococcal entry side into the CSF cannot be completely ruled out. In a previously established in vitro model of the BCSFB based on human choroidal epithelial cells, meningococci successfully invade and translocate through the epithelial layer, representing well the properties and barrier characteristics of the BCSFB [125,126,127]. A co-culture model based on human choroidal epithelial and endothelial cells would be an even better representation of the in vivo morphology of the BCSFB. Such a model would enable investigating the described snapshot during disease with meningococci located on and in choroidal endothelial cells and their way via the epithelial cells into the CSF.

As described above and depicted in Figure 1B, the BCSFB is formed by the epithelial cells of the choroid plexus that is located in the ventricular system. Noteworthy, bacteria interacting with the choroid plexus epithelium make contact first with the basolateral side of the epithelial cells after they have crossed the fenestrated choroid plexus endothelium.

**Figure 3:**
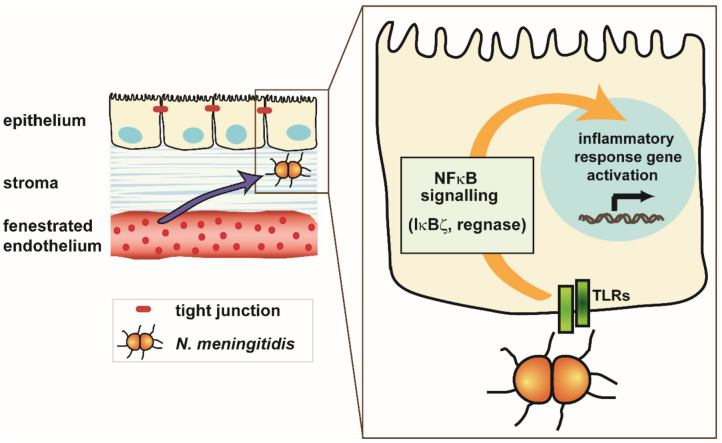
Interaction of *Neisseria meningitidis* with the choroid plexus epithelium. At the choroid plexus, the endothelial cells forming the capillaries are fenestrated and separated from the epithelium, which is tightly sealed by tight junctions, by a stroma. Meningococci reaching the epithelial cells interact with toll like receptors (TLRs), resulting in an NFκB-mediated activation of a pro-inflammatory immune response. NFκB signaling involves the transcription factor IκBζ as well as the ribonuclease regnase that mediates posttranscriptional regulation of inflammatory immune response genes.

Interactions of *N. meningitidis* with the choroid plexus epithelium are summarized in Figure 3. The choroid plexus are often attributed to be CNS sensors [13]. Performing their role thoroughly in response to bacterial, including meningococcal, infection, they secret pro-inflammatory cytokines into the CSF, thereby attracting immune cells such as neutrophils, a hallmark of bacterial meningitis [128,129,130]. Basolateral infection of human choroidal epithelial cells in vitro displays increased secretion of pro-inflammatory cytokines and chemokines including CXCL2, CXCL3, IL8, and IL6. Expression analysis of pattern recognition receptors (PRRs) reveals TLR2/TLR6 complex interactions with lipoprotein components of the bacterial cell wall more likely than TLR2/TLR1- or TLR4-mediated signaling. *Neisseria* internalization into epithelial cells in vitro also seems rather to involve TLR2/TLR1 than TLR4 signaling [131]. The PorB protein of *Neisseria* is considered as a TLR2/TLR6 ligand [132]. As shown with B cells from MyD88 and TLR2 knockout mice and cell lines in vitro, meningococcal PorB can directly interact with TLR2, leading to activation of immune cells via the TLR2/TLR1 complex and MyD88 dependent signaling [133,134]. In addition, a TLR2-dependent induction of IκBζ has been described [135], and *N. meningitidis* capsular polysaccharides induce inflammatory responses via TLR2 and TLR4-MD-2 in cell cultures in vitro [136].

Involvement of intracellular PRRs can also be considered for choroidal epithelial cells. PRR expression in the human plexus is still a secret, since it has never been analyzed. The only expression data of TLR2 and TLR4 are available from choroid plexus and meningeal vessels in mice and rat [137,138]. Receptor interactions in choroid epithelial cells in vitro result in an NFκB-mediated pro-inflammatory immune response involving the transcription factor IκBζ as well as regnase, a ribonuclease involved in posttranscriptional regulation of the inflammatory immune response [127,139]. NFκB-mediated as well as MAPK signaling involved in internalization has also been reported for bronchial epithelial cells [131].

Transmigrated meningococci have been observed in the in vitro model consisting of human choroidal epithelial cells in form of microcolinies at the apical cell side [125]. However, the putative signal transduction pathways in the transmigration process are unknown. Taken together, the choroid plexus is actively involved in meningitis pathogenesis, even though its role as a primary entry site may remain to be clarified.

#### 3.3.3. Neisserial Interactions with the Blood–Cerebrospinal Fluid Barrier at the Meninges

Bacterial meningitis predominantly represents an inflammation of the meninges, particularly the leptomeninges, composed of the pia mater and the arachnoid mater, with little or no involvement of the dura mater or the underlying brain parenchyma [19]. Thus, one hypothetical entry port into the CSF for meningococci, rather than the BBB, are veins in the SAS with endothelial walls, constituting the first line of barrier at the meningeal barrier, another component of the BCSFB (Figure 1C). The veins in the SAS are surrounded by a thin layer of connective tissue followed by a leptomeningeal lining that have to be penetrated to get access to the CSF-filled SAS [12].

Therefore, one focus of research is the investigation of meningococcal interactions with cells of the leptomeninges. Since the attempts to successfully culture primary human leptomeningeal cells failed [140], and in vivo animal models exhibit significant anatomical differences of the meninges compared to humans [19], two alternative models have previously been established, relying on cryostat sections of fresh human brain and cells cultured from benign tumors of the leptomeninges (meningiomas) [104]. Meningioma cells provide several benefits to cryostat sections and exhibit essential markers of leptomeningeal cells in vivo representing well the meningeal barrier [104,140].

Meningococci preferentially bind to leptomeningeal cells and meningeal blood vessels, but not to cortical brain tissue [104]. Meningococci similarly adhered to meningioma cells; however, no internalization could be observed [104], thus an intercellular crossing involving desmosomes appears more likely [12]. Several potential receptors binding meningococcal ligands can be postulated for leptomeningeal cells [12]. However, several well-known candidates including members of the TLR family, such as TLR2, TLR4, MD-2, or CD14, or LPS-accessory surface proteins HSP70 and HSP90a, chemokine receptor CXCR4, and growth differentiation factor (GDF) 5, seem not to be involved in the signaling upon meningeal meningococcal infection. Recognition of LPS and non-LPS modulins by meningeal cells more likely is dependent on the expression of yet uncharacterized PRRs [141].

Interaction of meningococci with meningioma cells in vitro leads to regulation of host cell genes, including genes for several pro-inflammatory and chemotactic cytokines and chemokines, such as TNFα, IL-6, IL-8, MIP-2α, and MCP-1, and genes for apoptosis-related factors including caspase-10, brain-derived neurotropic factor, IEX-1L anti-death protein, IRF-1, osteopontin, and the BB2-bombesin receptor [142]. It was furthermore shown that upon meningococcal infection meningioma cells specifically secrete pro-inflammatory (IL-6), chemotactic (IL-8, MCP-1, and RANTES), and growth factor-related (GM-CSF) cytokines [19,143]. Expression levels were affected by the presence of meningococcal surface components, such as Tfp, LPS, capsule, and possible secreted components [19]. A role of secreted proteins from *N. meningitidis* during differential gene expression and immune modulation in meningeal-derived cells was later confirmed. In those experiments, expression of IL-8, IL-6 ICAM-1, and COX-2 was shown [144]. Cytokines were also secreted when meningioma cells were challenged with the closely related organism *N. lactamica,* but levels of IL6 were significantly lower when cells were infected with *N. lactamica*.

Thus, leptomeningeal cells are actively involved in the innate immune response, secreting inflammatory cytokines, thereby contributing to the “inflammatory soup” of host molecules found in the CSF during leptomeningitis pathogenesis [12,19].

## 4. Conclusions

To enter the CNS, *N. meningitidis* has to cross the barriers between the blood and the brain, which are constituted by the BBB at the brain capillaries and the BCSFB at the choroid plexus and the meninges. At all of those barriers, meningococci undergo interactions with host cell factors, which lead to activation of signal transduction pathways. These pathways can contribute to plasma membrane remodeling during the adhesion and entry of the bacteria into the host cell, as well as to the regulation of the inflammatory response. However, further research is required to investigate in detail the interactions and involved signaling mechanisms at the different barriers. The disclosure of differences and commonalities will be very important to develop targeted strategies for treatment during the pathogenesis of meningococcal CNS infections.

## Figures and Tables

**Table 1:** Features common and differing in signaling pathways induced upon meningococcal attachment to epithelial and endothelial cells.

Nasopharyngeal Epithelium	Peripheral and Cerebral Endothelium
• Formation of cortical plaques [35,87,88] • Recruitment of ezrin [35,87,88] • Accumulation of actin [22,87] • Recruitment of adhesion molecules [35,87,88] • Recruitment of membrane receptors [35,87,88] • Recruitment of β2AR [76,88]	
No significant recruitment of the polarity complex (Par3/Par6) beneath colonies [76]	Recruitment of the polarity complex (Par3/Par6) beneath attached colonies colocalizing with cortical plaques [99]
No alteration delocalization and colocalization with ezrin under colonies of TJ (ZO1) and AJ proteins (p120 catenin, E-cadherin) [76]	Recruitment of junctional components (AJ: VE-cadherin, p120 catenin, and β-catenin; and TJ: ZO1, ZO2, and claudin-5) under the attached microcolonies [99]
Transcellular barrier crossing [22,34]	Formation of gaps between the cells promoting paracellular barrier crossing [99]
No activation of the β-arrestin pathway [76]	Activation of β-arrestin pathway upon adhesion [88]
β-arrestin signaling pathway independent formation of cortical plaques [76]	β-arrestin signaling pathway is essential for the formation of cortical plaques [88]
no involvement of Src kinase in cortical plaque formation and actin polymerization [76]	Recruitment of Src kinase below the attached colonies, Src involvement in formation of cortical plaques, and actin polymerization [76]
protrusions do not mediate protection against shear stress [76]	protrusions stabilize the colonies and protect them against blood flow generated shear stress [90]
induction of signaling is PilV independent [76]	Activation of the β-arrestin pathway is PilV (and PilE) dependent; Ezrin recruitment, cortical plaque, and shear stress resistance are PilV dependent [90]

## Abbreviations

AJ	Adherens junction
APC	Antigen presenting cell
ASM	Acid sphingomyelinase
β2AR	β2-adrenergic receptor
BBB	Blood–brain barrier
BCSFB	Blood–cerebrospinal fluid barrier
CEACAM	Carcinoembryonic antigen-related cell adhesion molecules
CNS	Central nervous system
CSF	Cerebrospinal fluid
CVO	Circumventricular organ
ECM	Extracellular matrix
FAK	Focal adhesion kinase
GDF	Growth differentiation factor
GLUT1	Glucose transporter 1
HSPG	Heparin sulfate proteoglycan
IMD	Invasive meningococcal disease
JAM	Junction adhesion molecule
LPS	Lipopolysaccharide
MAGUK	Membrane-associated guanylate kinase
MAPK	Mitogen-activated kinase
MARVELD3	MARVEL domain-containing protein 3
MHC	Major histocompatibility complex
MLST	Multi locus sequence typing
MMP	Matrix metalloprotease
MRT	Magnetic resonance tomography
*N. meningitidis*	*Neisseria mengitidis*
ORF	Open reading frame
PC-PLC	Phosphatidylcholine specific phospholipase C
pdhC	Pyruvate dehydrogenase subunit
PECAM	Platelet endothelial cell adhesion molecule
PLVAP	Plasmalemma vesicle-associated protein
Pgp	P-glycoprotein
PRR	Pattern recognition receptor
SAS	Subarachnoidal space
SLC	Solute carrier
ssm	Single strand mispairing
TAMP	Tight junction–associated MARVEL protein
TEER	Transepithelial electrical resistance
TJ	Tight junction
Tfp	Type IV pilus
TLR	Toll like receptor
VE-cadherin	vascular endothelial cadherin
VEGF	Vascular endothelial growth factor
ZO	Zonula occludens
